# Two isothermal challenges yield comparable physiological and subjective responses

**DOI:** 10.1007/s00421-020-04494-3

**Published:** 2020-09-20

**Authors:** L. Klous, A. Psikuta, K. Gijsbertse, D. Mol, M. van Schaik, H. A. M. Daanen, B. R. M. Kingma

**Affiliations:** 1 Faculty of Behavioural and Movement Sciences, Vrije Universiteit Amsterdam, Amsterdam, The Netherlands; 2 TNO, Netherlands Organization for Applied Scientific Research, Defense, Safety & Security, Soesterberg, Kampweg 55, 3769 DE Soesterberg, The Netherlands; 3 Empa, Swiss Federal Laboratories for Materials Science and Technology, St. Gallen, Switzerland; 4 Department of Nutrition, Exercise and Sports, Section for Integrative Physiology, University of Copenhagen, Copenhagen, Denmark; 5 Department of Energy Technology, Eindhoven University of Technology, Eindhoven, The Netherlands

**Keywords:** Ventilated vest, Thermal resistance, Thermal stress, Local sweat rate

## Abstract

**Purpose:**

Ventilated vests are developed to reduce thermal stress by enhancing convective and evaporative cooling from skin tissue underneath the vest. The purpose of this study is to investigate whether thermal stress is equal when a ventilated vest is worn compared to a no-vest situation with similar dry thermal resistance.

**Methods:**

Nine healthy males walked on a treadmill (7 km h^−1^) for 45 min in a desert climate (34 °C, 20% relative humidity) with and without ventilated vest. Gastrointestinal temperature (*T*_gi_), heart rate (HR), and skin temperature (*T*_sk_) were continuously monitored. Local sweat rate (LSR) was assessed two times on six skin locations. Subjective ratings were assessed every 10 min.

**Results:**

Final *T*_gi_ (37.6 ± 0.1 °C for vest and 37.6 ± 0.1 °C for no-vest), HR (133 ± 7 bpm and 133 ± 9 bpm) and mean T_sk_ (34.8 ± 0.7 °C and 34.9 ± 0.6 °C) were not different between conditions (*p* ≥ 0.163). Scapula skin temperature (*T*_scapula_) under the vest tended to be lower (baseline to final: Δ*T*_scapula_ = 0.35 ± 0.37 °C) than without vest (Δ*T*_scapula_ = 0.74 ± 0.62 °C, *p* = 0.096). LSR at locations outside the vest did not differ with and without vest (*p* ≥ 0.271). Likewise, subjective responses did not differ between conditions (*χ*^2^ ≥ 0.143).

**Conclusions:**

We conclude that two systems with similar dry thermal resistance and, therefore, similar required evaporation, resulted in similar thermal stress during paced walking in a hot-dry environment. Local ventilation did not alter the sweating response on locations outside the vest.

## Introduction

Ventilated vests are developed to reduce thermal stress by enhancing convective and evaporative cooling from skin tissue underneath the vest. By enhancing convection and evaporation, the exercise-induced increase in body core temperature may be attenuated (Barwood et al. 2009; Reffeltrath 2006). Even without a physiological effect, if a ventilated vest can reduce discomfort by providing a lower heat sensation this may also be considered beneficial. As previous research showed that local discomfort overrides whole-body comfort (Zhang et al. 2004), a ventilated vest could positively influence whole-body subjective comfort or thermal sensation by mitigating local discomfort. The other way around, it is discussed whether a local warm stimulus can be detrimental to performance through psychological stress (i.e., thermal sensation) (Lloyd and Havenith 2019; Van Cutsem et al. 2019). In the experiment of Van Cutsem et al. (2019), twelve trained cyclists or triathletes performed a time to exhaustion test which consisted of a warming-up (cycling for 5 min at 40% VO_2max_), followed by cycling until exhaustion at 70% VO_2max_ in a climate chamber set to 20 °C and 44% relative humidity (RH). Their participants performed the trial twice: one time with and one time without a 30 × 40 cm electric heat pad (~ 40 °C) covering the upper back and both scapulae. Time to exhaustion was found to decrease by 9% without changes in physiological measures. Thus, the performance decrease was attributed to an increased subjective thermal strain (Van Cutsem et al. 2019). However, Lloyd and Havenith (2019) argued that the actual heat load was increased by 4–6% in the experiment of Van Cutsem et al. (2019) so it could have been that there was no independent role of subjective thermal strain in the heat-induced reduced performance. Here we are interested in whether two different local clothing setups, which are comparable with respect to physical thermal strain, would result in comparable physiological, in particular local sweating, and subjective thermal responses.

The human body exchanges heat through conduction, convection, radiation (dry heat exchange) and evaporation (latent heat exchange). In common work situations, the body’s maximal cooling capacity is limited by convective and evaporative heat loss capacity (Kenney et al. 2015). Convective heat loss is a function of the effective air velocity over the body surface area and the temperature gradient between the air and the body surface (Parsons 2014). Evaporative heat loss is a function of the amount of sweat that is produced and the physical ability of sweat to evaporate (Parsons 2014). That is, in the best cooling scenario the sweating response is maximal and all sweat can evaporate. This is most likely to occur in situations with a high vapour pressure difference between skin and air (i.e., with a low RH) and high air speed. As ambient temperature increases, the effectiveness of convection to facilitate body heat loss decreases due to the small temperature gradient between air and skin temperature (*T*_sk_). Consequently, in hot environments evaporation provides the primary defense against overheating (Parsons 2014). A variety of external cooling strategies to lower the exercise-induced increase in body core temperature by enhancing convection and evaporation, including ventilated vests, have been evaluated in the military, occupational settings and elite sports (Barwood et al. 2009; Bongers et al. 2017; Eijsvogels et al. 2014; O’Hara et al. 2016). Prior to using such systems in occupational settings or sports, its performance should ideally be assessed using standard methods (ASTM-F2300-10 2016; ASTM-F2371-16 2010).

From a physiological control point of view, body core temperature is the central controller for the production of sweat and *T*_sk_ has a modifying effect (McCaffrey et al. 1979; Nadel et al. 1971a, b). This modifying effect of *T*_sk_ is estimated to be a tenth of the core temperature part (Nadel et al. 1971a). The effect is stimulating when *T*_sk_ is high and inhibiting when *T*_sk_ is low. By applying local ventilation using a ventilated vest, local *T*_sk_ may decrease, causing less sweat production underneath the vest. However, recently Hospers et al. (2020) showed that whole-body sweat rate is highly correlated to required evaporation (*E*_req_) and does not depend on mean *T*_sk_ independent of *E*_req_ to achieve heat balance. From a biophysical perspective, *E*_req_ is defined by body heat production minus dry heat loss from skin and respiratory heat loss (Cramer and Jay 2016). Since the aim of our study is to create two locally isothermal situations, no differences in local *E*_req_ and consequently local sweat rate (LSR) is expected, as per heat balance theory. Note that sweating per se is not equal to evaporation, and maximal evaporation (*E*_max_) is limited by the vapour pressure difference between skin tissue and the air, and the convective heat transfer coefficient (Cramer and Jay 2016). With respect to a ventilated vest, it is unknown whether local enhancement of evaporation would cause a decrease in local required evaporation on other skin sites (to balance the global *E*_req_). If true, LSR (supply of water) outside the ventilated vest should decrease proportionally to the amount of sweat that is evaporated extra underneath the vest.

Ventilated vests come with the cost of extra insulation. However, by bridging the low conductivity of the vest with ventilation one can compensate for insulation or even exceed it if applying even higher ventilation rates. In the current study, two isothermal systems were tested: one with and one without ventilated vest. The air speed of the ventilated vest was set such that it compensated for the dry thermal resistance of the vest to create two different but isothermal challenges. It was hypothesized that two systems with similar dry thermal resistance—and, therefore, similar *E*_req_—provide similar thermal stress, measured by physiological and subjective responses. If successful, the present study provides a method to calculate the limit of air movement (due to walking) for which a ventilated vest is beneficial compared to wearing no ventilated vest, or the required ventilation for protective equipment in occupational settings. This may be useful to extend the verification procedure for standard settings on ventilated vest performance (ASTM-F2300-10 2016; ASTM-F2371-16 2010; McCullough and Eckels 2009).

## Methods

### Ethical approval

Procedures were approved by the local Ethics Committee of TNO (report number 2018-054). Procedures used in this study adhere to the tenets of the Declaration of Helsinki, except for registration in a database. Upon arriving at the laboratory, participants signed informed consent and filled out a basic health check.

### Participants

Contrary to an a priori sample size analysis to detect a difference between two or more (in)dependent means, this study was set up to detect only large effect sizes. The rationale behind this logic is that if the difference cannot be clearly detected in a small group of participants, it may not be physiologically important in an operational setting (military squad size). Given *α* = 0.05, power = 0.8 and *n *= 10, we were able to detect a difference between two dependent means once the effect size exceeded 0.85. Ten recreationally trained male participants were recruited based on anthropometrics to fit the ventilated vest. Nine participants (age: 24.5 ± 2.2 years, weight: 84.8 ± 3.0 kg, height: 189.4 ± 3.6 cm, body mass index: 23.9 ± 2.5 kg m^−2^, training frequency: ≥ 3 times 1.5 h per week) completed the experimental protocol.

### Design

All participants performed the same exercise protocol twice with at least 48 h in between to prevent heat acclimation (Gill and Sleivert 2001). On one occasion, participants walked for 45 min at 7 km h^−1^ on a treadmill (Bari Mill 55, Woodway, Waukesha, Wisconsin, USA) in a hot-dry environment (34 °C, 20% RH) in a climate chamber (Weiss Technik, Tiel, The Netherlands) while wearing the ventilated vest. On a second occasion, they did the exact same without wearing the ventilated vest. The order of tests was randomized. The remaining procedure was similar for both days.

Participants were asked to avoid intense exercise, refrain from alcohol and caffeine 24 h before the test and were asked to drink specified amounts of water to ensure euhydration (Sawka et al. 2007). Approximately 1 h prior to the session in the climate chamber, participants ingested a capsule for measurement of gastrointestinal temperature (*T*_gi_) (BodyCap, e-Celsius Performance, Hérouville Saint-Clair, France). Previous research showed a gastric residence time of 1.5–3.5 h after ingesting an indigestible capsule 30 min after a liquid meal and 1.5–7 h after a solid meal (Mojaverian et al. 1985). As participants in the current study drunk 500 ml of water before reporting to the laboratory as prescribed by the hydration protocol, gastric residence time was estimated to be more than 1 h. If participants also ate a solid meal before reporting to the laboratory, gastric residence time was even longer. Therefore, the capsule was most likely located in the stomach when participants started the experiment (± 1 h after ingestion), but the capsule could have entered the duodenum during the experiment (Mojaverian et al. 1985). Consequently, participants were equipped with a heart rate monitor (V800, Polar, Kempele, Finland) and four temperature sensors (iButton, Maxim Integrated, San Jose, California, USA) located at the neck (*T*_neck_), right scapula (*T*_scapula_), left hand (*T*_hand_) and right shin (*T*_shin_) (ISO9886 2004). Mean *T*_sk_ was calculated according to ISO 9886. The temperature sensor on the scapula was the only sensor located underneath the vest.

Thereafter, participants wore a merino wool shirt (Aclima Safe Net, size M, 75% Merino Wool, 15% Para-Aramide, 10% Polyamide) with the ventilated vest and battle dress uniform (BDU) (Marquardt and Schulz, size L, 65% Cotton, 35% Polyester) on top. After entering the climate chamber (34 °C, 20% RH), participants sat on a chair for 15 min, followed by a 45-min walk at 7 km h^−1^ on a treadmill without being allowed to drink. They were asked to indicate their rating of perceived exertion (RPE), thermal sensation (TS) and thermal comfort (TC) every 10 min. RPE was assessed with a 15-point scale (ranging from 6 rest to 20 maximal exercise) (Borg 1982), TS with a nine-point scale (− 4 very cold; − 3 cold; − 2 cool; − 1 slightly cool; 0 neutral; + 1 slightly warm; + 2 warm; + 3 hot; + 4 very hot) (ISO10551 2001), and TC with a five-point scale (1 comfortable; 2 slightly uncomfortable; 3 uncomfortable; 4 very uncomfortable; 5 extremely uncomfortable) (ISO10551 2001).

LSR was determined using the absorbent patch technique (Morris et al. 2013). Absorbent patches (3 M Tegaderm + Pad, Maplewood, Minnesota, USA, size: 10 cm^2^, absorbing capacity: ~ 1.3 g) were attached to the central and peripheral part of the body for 10 min. Sweat patches covered zone 12–13 (central upper back), zone 1 (forehead), zone 19 (both upper arms) and zone 25 (both upper legs) of the human body as described by Gerrett et al. (2014). Sweat samples were taken from both left and right extremities because of the large regional differences (Smith and Havenith 2011). After 10 min of sampling, participants had to stop walking for 1 min to replace the absorbent patches. Patches were weighed before and right after application (1419MP8-1, Sartorius, Göttingen, Germany) to calculate LSR according to the following formula:

$${\text{LSR}} = ((m_{\text{wet}} - m_{\text{dry}} )/{\text{SA}})/t,$$

where ‘$m$_wet_’ refers to the mass of the wet patch (g) after the experiment, ‘$m$_dry_’ to the dry mass of the patch (g), ‘SA’ is surface area of the absorbent patch (m^2^) and ‘*t*’ represents the application in hours. LSR is given in gm^−2^ h^−1^. The determination of LSR was done from min *t*_25_–*t*_35_ and *t*_35_–*t*_45_ of walking.

Before and right after the session, weight of the fully equipped participants and subsequently of the clothing was taken (F300S, Sartorius, Göttingen, Germany). Evaporated sweat mass was determined as the difference between the weight of fully equipped participants and their clothing before and after the session in the climate chamber. Whole-body sweat loss (WBSL) was calculated by adding the mass of accumulated sweat in the clothing (mass difference clothing pre- and post-exercise) to evaporated mass.

### Ventilated vest

**Fig. 1:**
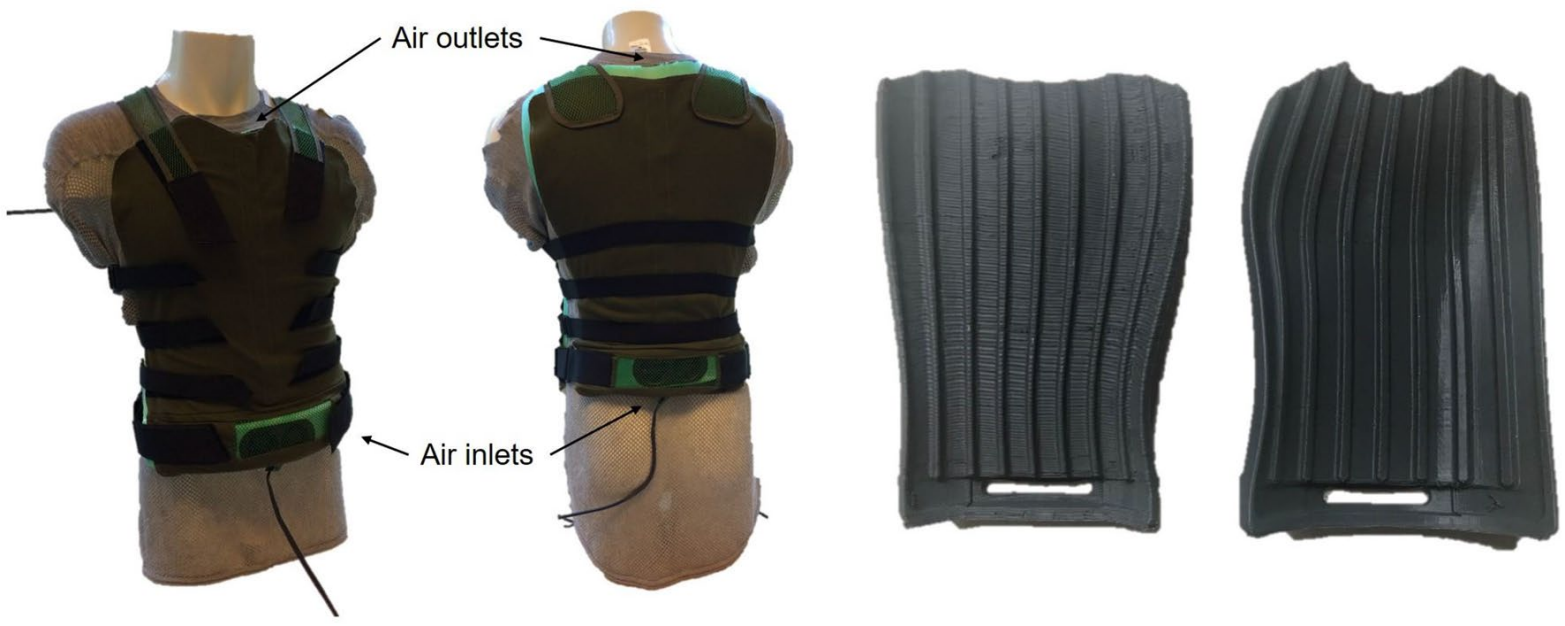
Ventilated vest. Ventilators at the air inlets allow for the flow of air through channels in the vest. On the left, the vest is shown from the outside, on the right the vest is shown from the inside. The ventilated vest and battle dress uniform (BDU), respectively, were worn on top of the merino wool shirt

**Fig. 2:**
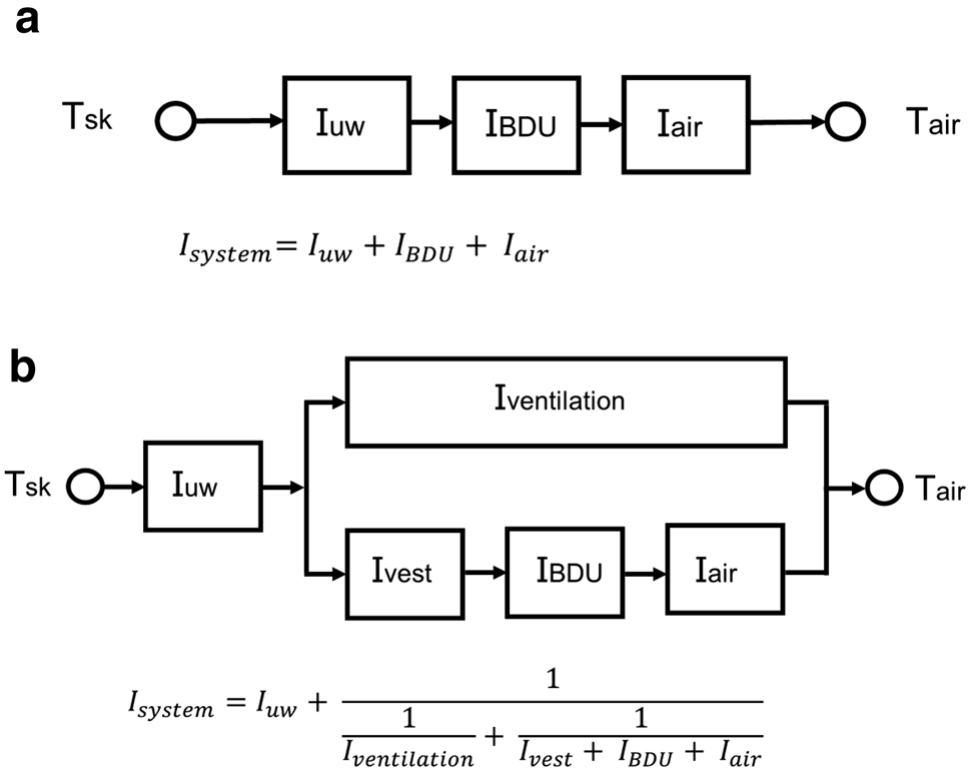
Thermal network. Typical thermal situation, where the heat from the human body (shown with the arrows) must transfer from the skin (*T*_sk_) through underwear, battle dress uniform (BDU) and boundary air layers towards the environment (*T*_air_) (**a**). Introduction of a ventilated vest directly connects the circulating air underneath the vest to the environment (**b**). Thermal insulation of the system ($I_{\text{system}}$) is calculated according to the formulas below the thermal network. $I_{\text{uw}}$, $I_{\text{ventilation}}$, $I_{\text{vest}}$, $I_{\text{BDU}}$ and $I_{\text{air}}$ represent thermal insulation of the underwear, ventilation, ventilated vest, BDU and air, respectively

Ventilation was achieved by blowing ambient air through parallel channels indented on the inner side of the vest made of polyurethane foam and tightened through contact with the body (merino wool shirt in between) (Fig. 1). The air movement direction was from bottom to top and was induced by four 405FH Ebm-papst fans (5 V, 0.18A, 0.9 W, Mulfingen, Germany) mounted at the bottom of the vest. The fans allowed to achieve an air speed of 1.5 ms^−1^ underneath the vest. Weight of the ventilated vest, including batteries, was 1.3 kg. By design, the ventilated vest circumvents resistance that was provided by insulating layers. In a typical situation, the human body heat must transfer from the skin through clothing towards the environment (Fig. 2a). However, using the vest, circulating air underneath the vest was directly connected to the environment. The ventilated vest created a shortcut for heat to bypass all layers of thermal resistance on top of it (Fig. 2b).

The aim of the current study was to create two different isothermal settings: one with and one without ventilated vest underneath a BDU. By bridging the low conductivity of the vest with ventilation, we compensated for its insulation. To evaluate if the thermal resistance of the ventilated vest underneath a BDU and BDU alone was similar, the vest was tested on a Sweating Agile thermal Manikin (SAM) (Psikuta 2009; Richards and McCullough 2005). SAM is a multi-segmental and anatomically formed thermal sweating manikin. Mean temperature of each shell part is measured at its outer surface using nickel resistance wire. Local thermal resistance values of the torso (back and chest) were determined according to the ISO9920 parallel method (ISO9920 2007) with manikin surface temperature kept constant at 34 °C and the environmental conditions at 20 °C, 50% RH and < 0.2 ms^−1^ wind speed applied from the front and back. Local torso values, rather than whole-body values, were measured because the manipulation (i.e., ventilated vest) was applied locally. All other parts of the clothing ensemble were unchanged—and equal—between conditions. Thermal insulation of individual layers was measured independent of the system because of the potential translation to other combinations of clothing ensembles. An example of adding a ballistic and load-bearing vest is given in the discussion. Unlike all other determinations, while measuring thermal insulation of the underwear wind was applied from the front only. The local clothing area factor ($f_{{\text{cl}}}$) indicates the increase in surface area of heat loss by adding clothing. In both conditions, thermal insulation of the air layer around the manikin ($I_{{\text{air}}}$) should be divided by $f_{{\text{cl}}}$ of the outer layer of the ensemble (i.e., BDU) to account for this increase (Vesela et al. 2018). $f_{{\text{cl}}}$ of the outer layer of the BDU on the torso was determined by making use of its linear relation with ease allowance (Vesela et al. 2018). Ease allowance, which is the difference between circumferences of a clothing item and the manikin, was measured.

In a static situation, thermal insulation (local torso values) was 0.029 m^2^ KW^−1^ for the underwear ($I_{\text{uw}}$), 0.157 m^2^ KW^−1^ for BDU ($I_{\text{BDU}}$) with $f_{\text{cl,BDU}}$ = 1.19, and 0.116 m^2^ KW^−1^ for the air ($I_{\text{air}}$) resulting in $I_{\text{system}}$ = 0.283 m^2^ KW^−1^ local clothing insulation in the no-vest condition (Fig. 2a, Table 1). In the vest condition (ventilation speed 1.5 ms^−1^), estimated thermal insulation of the underwear decreased to 0.020 m^2^ KW^−1^. Measured insulation of the ventilated vest ($I_{\text{vest}}$), ventilation ($I_{\text{ventilation}}$) and $I_{\text{air}}$ at an air speed of 1.5 ms^−1^ were 0.304 m^2^ KW^−1^, 0.264 m^2^ KW^−1^ and 0.116 m^2^ KW^−1^, respectively, with $f_{\text{cl,vest + BDU}}$ = 1.69. Here, $f_{\text{cl}}$ was higher because the surface area for heat loss increased by adding the ventilated vest underneath BDU. This results in $I_{{{\text{system,1}} . 5}}$ = 0.196 m^2^KW^−1^ in the vest condition (Fig. 2b, Table 1). Hereafter, $I_{\text{BDU}}$ and $I_{\text{air}}$ were corrected for the effects of body movement (paced walking at 7 km h^−1^) according to (ISO9920 2007):

$$I_{{{\text{BDU}},r}} = e^{{\left[ { - 0.281\left( {v_{\text{air}} - 0.15} \right) + 0.044(v_{\text{air}} - 0.15)^{2} - 0.492v_{w} + 0.176v_{w}^{2} } \right]}} I_{\text{BDU}} ;$$

$$I_{{{\text{air}},r}} = e^{{\left[ { - 0.533\left( {v_{\text{air}} - 0.15} \right) + 0.069(v_{\text{air}} - 0.15)^{2} - 0.462v_{w} + 0.201v_{w}^{2} } \right]}} I_{\text{air}}$$

$I_{{{\text{BDU}},r}}$ and $I_{{{\text{air}},r}}$ represent the resultant thermal insulation after correction for the effects of body movement, $v_{\text{air}}$ represents air speed relative to the person (0.15 ms^−1^) and $v_{w}$ walking speed (1.944 ms^−1^). Therefore, $I_{{{\text{BDU}},r}}$ = 0.074 m^2^ KW^−1^ and $I_{{{\text{air}},r}}$ = 0.101 m^2^ KW^−1^, resulting in $I_{{{\text{system}},r}}$ = 0.188 m^2^ KW^−1^ for the no-vest condition, which can be compared to the vest condition: $I_{{{\text{system}},r,1.5}}$ = 0.185 m^2^ KW^−1^ (Table 1).

**Table 1:** Measured thermal insulation of the underwear ($I_{\text{uw}}$), battle dress uniform ($I_{\text{BDU}}$), air ($I_{\text{air}}$), ventilated vest ($I_{\text{vest}}$), ventilation ($I_{\text{ventilation}}$) and total system ($I_{\text{system}}$) in the vest and no-vest condition

	Vest			No-vest		
	Static	$f_{\text{cl}}$	7 km h^−1^	Static	$f_{\text{cl}}$	7 km h^−1^
$I_{\text{uw}}$ (m^2^KW^−1^)	0.020	–	0.020	0.029	–	0.029
$I_{\text{ventilation}}$ (m^2^KW^−1^)	0.304	–	0.304	–	–	–
$I_{vest}$ (m^2^KW^−1^)	0.264	–	0.264	–	–	–
$I_{\text{BDU}}$ (m^2^KW^−1^)	0.157	1.69	0.074	0.157	1.19	0.074
$I_{\text{air}}$ (m^2^ KW^−1^)	0.116	–	0.101	0.116	–	0.101
$I_{\text{system}}$ (m^2^ KW^−1^)	0.196	–	0.185	0.283	–	0.188

Results are presented for a static and dynamic (walking at 7 km h^−1^; resultant thermal insulation) situation, of which the latter represents the situation in the current study. In the vest condition, ventilation speed was set at 1.5 ms^−1^ in both the static and dynamic situations. Corresponding clothing area factors ($f_{\text{cl}}$) are presented where appropriate (i.e., the outer layer of the clothing ensemble). To calculate $I_{\text{system}}$, $I_{\text{air}}$ should be divided by $f_{\text{cl, BDU}}$ to correct for the increase in surface area for heat loss

### Data analysis

Baseline *T*_gi_, HR, *T*_neck_, *T*_scapula_, *T*_hand_ and *T*_shin_ values were obtained during the first 5 min of sitting in the climate chamber. Final *T*_gi_, HR, *T*_neck_, *T*_scapula_, *T*_hand_ and *T*_shin_ values were obtained during the final 5 min of walking in the climate chamber. The assumption of normally distributed differences between pairs was tested using histograms and the Kolmogorov–Smirnov test. To test for significant (*p* < 0.05) differences in mean physiological responses in the vest and no-vest condition, a repeated measures ANOVA was used (SPSS Statistics 26, IBM, USA). Subjective data are ordinal, hence, the non-parametric Friedman test was performed. Effect sizes (*η*^2^ for parametric data and Kendall’s W for non-parametric data) were calculated with the following criteria: < 0.20 is classified as trivial, 0.21–0.49 as small, 0.50–0.79 as moderate and > 0.80 as a large effect (Cohen 1988). Data are presented as mean and standard deviation (SD).

## Results

### Evaporated mass, whole-body sweat loss, gastrointestinal temperature and heart rate

**Fig. 3:**
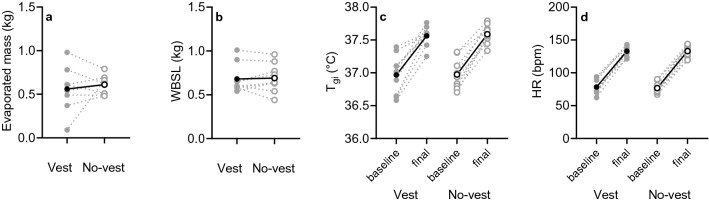
Scatterplots for evaporated mass (**a)**, whole-body sweat loss (WBSL) (**b**), baseline (5-min sit) and final (final 5 min of walking) gastrointestinal temperature (*T*_gi_) (**c)**, baseline and final heart rate (HR) (**d**) in the vest (cycles) and no-vest condition (empty circles). The solid line and black circle represent mean data (*n *= 9), whilst the grey dashed line and circles are the individual responses

One participant could not attend both occasions due to illness and was excluded from the study. Figure 3 shows the evaporated mass, WBSL, *T*_gi_ (baseline to final) and HR (baseline to final) in the vest and no-vest conditions. All of these variables did not differ between conditions (evaporated mass: *p* = 0.531, WBSL: *p* = 0.516, *T*_gi_: *p* ≥ 0.510, HR: *p* ≥ 0.160). Table 2 shows the repeated measures ANOVA results for evaporated mass, WBSL, *T*_gi_ and HR.

**Table 2:** Overview of the repeated measures ANOVA results for evaporated mass, whole-body sweat loss (WBSL), gastro-intestinal temperature (*T*_gi_), heart rate (HR), and neck, scapula, hand and shin skin temperature (*T*_neck_, *T*_scapula_, *T*_hand_, *T*_shin_)

	Baseline		Final		Δ	
	*p* value	*η*^2^	*p* value	*η*^2^	*p* value	*η*^2^
Evaporated mass (kg)	**–**	**–**	**–**	**–**	0.531	0.051
WBSL (kg)	**–**	**–**	**–**	**–**	0.516	0.055
*T*_gi_ (°C)	0.837	0.005	0.510	0.050	0.696	0.020
HR (bpm)	0.160	0.207	0.945	0.001	0.504	0.058
*T*_neck_ (°C)	0.077	0.308	0.944	0.001	0.359	0.106
*T*_scapula_ (°C)	0.450	0.073	0.322	0.122	0.096	0.307
*T*_hand_ (°C)	0.398	0.080	0.616	0.029	0.582	0.039
*T*_shin_ (°C)	0.571	0.037	0.215	0.165	0.582	0.039

Baseline indicates the average of 5 min of sitting in the climate chamber, final indicates the average of the final 5 min of walking and Δ indicates the change from baseline to final. *η*^2^, measure of effect size

### Skin temperature

**Fig. 4:**
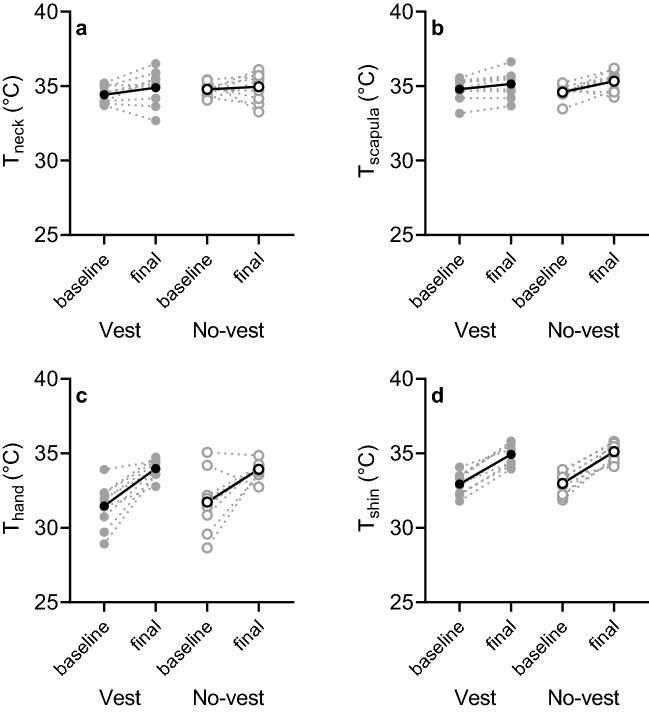
Scatterplots for baseline (5-min sit) and final (final 5 min of walking) neck skin temperature (*T*_neck_) (**a**), scapula skin temperature (*T*_scapula_) (**b**), hand skin temperature (*T*_hand_) (**c**) and shin skin temperature (*T*_shin_) (**d**) in the vest (cycles) and no-vest condition (empty circles). The solid line and black circle represent mean data (*n *= 9), whilst the grey dashed line and circles are the individual responses

Figure 4 shows *T*_neck_, *T*_scapula_, *T*_hand_ and *T*_shin_ (baseline to final) in the vest and no-vest condition. No significant differences were observed (*T*_neck_: *p* ≥ 0.077, *T*_scapula_: *p* ≥ 0.096, *T*_hand_: *p* ≥ 0.398, *T*_shin_: *p* ≥ 0.215). Table 2 shows the repeated measures ANOVA results for *T*_neck_, *T*_scapula_, *T*_hand_ and *T*_shin_.

### Local sweat rate

**Fig. 5:**
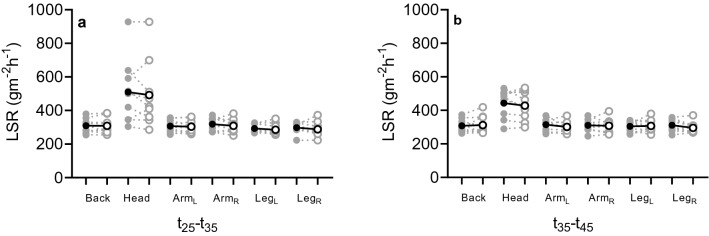
Scatterplots for local sweat rate (LSR) for *t*_25_–*t*_35_ (**a**) and *t*_35_–*t*_45_ (**b**) on the central upper back, forehead, left upper arm (Arm_L_), right upper arm (Arm_R_), left upper leg (Leg_L_) and right upper leg (Leg_R_) in the vest (cycles) and no-vest condition (empty circles). The solid line and black circle represent mean data (*n *= 9), whilst the grey dashed lines and circles are the individual responses

Figure 5 shows scatterplots for the non-significant differences in LSR at six skin locations (central upper back: *p* ≥ 0.560, forehead: *p* ≥ 0.933, both upper arms: *p* ≥ 0.421, and both upper legs: *p* ≥ 0.271) outside the vest in the vest and no-vest condition. In addition, Table 3 lists the repeated measures ANOVA results for LSR.

**Table 3:** Overview of the repeated measures ANOVA results for local sweat rate (LSR) at six measurement locations (central upper back, forehead, both upper arms and both upper legs) for *t*_25_–*t*_35_ and *t*_35_–*t*_45_

LSR (gm^−2^ h^−1^)	25–35 min		35–45 min	
	*p* value	*η*^2^	*p* value	*η*^2^
Back	1.000	0.000	0.560	0.044
Forehead	0.957	0.000	0.933	0.001
Arm_L_	0.890	0.003	0.421	0.082
Arm_R_	0.607	0.034	0.899	0.002
Leg_L_	0.271	0.149	0.720	0.017
Leg_R_	0.821	0.007	0.356	0.107

Arm_L_, left arm; Arm_R_, right arm; Leg_L_, left leg; Leg_R_, right leg; *η*^2^, measure of effect size

### Subjective responses

**Fig. 6:**
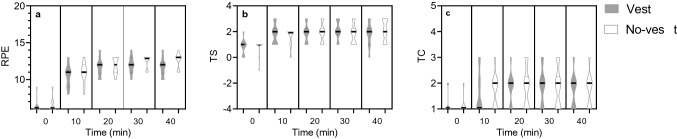
Violin plots of rating of perceived exertion (RPE) (**a**), thermal sensation (TS) (**b**) and thermal comfort (TC) (**c**) over the time course of the experiment in the vest (solid violins) and no-vest condition (empty violins). The horizontal black line represents the median (*n *= 9)

No statistically significant differences were found between the vest and no-vest condition for RPE (*χ*^2^ ≥ 0.143), TS (*χ*^2^ ≥ 0.333) and TC (*χ*^2^ ≥ 0.200; Fig. 6). Table 4 shows the Friedman’s ANOVA results at 10-min time intervals.

**Table 4:** Overview of the Friedman’s ANOVA results for rating of perceived exertion (RPE), thermal sensation (TS) and thermal comfort (TC) at *t*_0_, *t*_10_, *t*_20_, *t*_30_ and *t*_40._ *χ*^2^, test statistic; *W*, measure of effect size

	*t*_0_		*t*_10_		*t*_20_		*t*_30_		*t*_40_	
	*χ*^2^	*W*	*χ*^2^	*W*	*χ*^2^	*W*	*χ*^2^	*W*	*χ*^2^	*W*
RPE	3.000	0.333	0.143	0.016	1.000	0.111	0.667	0.074	2.000	0.222
TS	0.333	0.037	1.000	0.111	1.000	0.000	2.000	0.222	1.000	0.111
TC	1.000	0.111	0.200	0.022	1.000	0.000	0.200	0.022	1.000	0.000

## Discussion

This study shows that two clothing systems, one of them with a highly insulating ventilated vest, but both with similar local dry effective thermal resistance (0.185 m^2^ KW^−1^ for the ventilated vest condition and 0.188 m^2^ KW^−1^ for no-vest) provided similar thermal stress. No significant differences between conditions were found in evaporated mass, WBSL, *T*_gi_, and HR throughout the sessions in the climate chamber. In addition to these central thermal responses, no significant differences were found in *T*_neck_, *T*_scapula_, *T*_hand_ and *T*_shin_ between conditions. Likewise, LSR on the central upper back, forehead, upper arms and upper legs was not significantly different between conditions. Subjective responses supported these findings as no significant differences were found between the vest and no-vest condition in RPE, TS and TC.

These findings add to a recent debate discussing whether a local warm stimulus would cause a shorter time to exhaustion in a temperate condition (20 °C, 44% RH) (Lloyd and Havenith 2019; Van Cutsem et al. 2019). One of the main points of discussion was whether the local warm stimulus added true physical heat stress or only psychological heat stress (i.e., thermal sensation) as the authors argued. The authors found a 9% decrease in time to exhaustion without changes in physiological measures. They therefore attributed the performance decrease to an increased subjective thermal strain. However, Lloyd and Havenith (2019) suggested that heat load was increased by 4–6% in their experiment (Lloyd and Havenith 2019). In this paper, we show that in two different but isothermal settings no physiological and subjective differences were observed during a fast-paced walking activity (Figs. 3, 4, 5, 6). While this study did not provide a local warm stimulus and compensated with local cooling at a different site, we do show that in absence of a physical difference in heat stress, no difference in thermal outcome measures can be expected.

The skin temperature sensor on the scapula was the only sensor located underneath the vest, but there was no significant decrease in *T*_scapula_ in the vest condition. There was a trend towards lower values: Δ*T*_scapula_ = 0.35 ± 0.37 °C from baseline to final with vest and Δ*T*_scapula_ = 0.74 ± 0.62 °C (*p* = 0.096) in the no-vest condition (Fig. 4b). Considering the minor contribution of *T*_sk_ to sweating (Nadel et al. 1971a, b) and the limited changes in *T*_scapula_, this most likely explains why LSR was not affected by the ventilated vest either (Fig. 5). In combination with the comparable core temperature (as indicated by *T*_gi_; Fig. 3), the main driver of sweat production (Nadel et al. 1971a, b), it is not surprising that LSR was similar between conditions as per physiological control point of view. Our findings also support the biophysical perspective on the regulation of sweating. Since on the torso the ventilated vest manipulation led to equal thermal strain compared to a no-vest situation (Table 1), presumably no differences in *E*_req_ occurred between conditions (Gagnon et al. 2013; Ravanelli et al. 2020). With similar *E*_req_ values, the absence of differences in LSR can likely be explained. Interestingly, on skin locations outside the ventilated vest, the underwear (i.e., on the upper arms) was slightly wet. On these locations, the residual local *E*_req_ potentially was higher than the local *E*_max_. The absence of a difference in LSR on the upper arm (Fig. 5) may be contributed to achieving the local *E*_max_. On skin locations under the vest, it is unlikely that *E*_max_ was achieved as underwear was dry underneath the ventilation channels. Another possible explanation for the similar LSR could be that in addition to thermal factors, sweating is influenced by exercise-induced non-thermal factors including positive stimuli from mechanoreceptors and metaboreceptors (Kondo et al. 2010). Since the same exercise protocol was utilized in the two isothermal conditions, the non-thermal contribution to sweating was most likely similar. Depending on how big the contribution of non-thermal factors was, this could help in explaining our findings.

In the current study, large individual variation between physiological and subjective responses was observed (Figs. 3, 4, 5, 6), which may be explained by differences in personal characteristics such as training background. Fitness level and body composition were assumed to be homogenous considering the small range in body mass index, the inclusion of participants with a certain training frequency (≥ 3 times 1.5 h a week) and appropriate anthropometrics to fit the ventilated vest (i.e., little upper body fat) and were therefore not expected to influence our results. The large regional variation in LSR that was found in the current study is in accordance with existing literature (Smith and Havenith 2011) and the absorbent patch technique used in the current study correlates well with measurements of a ventilated capsule system (Morris et al. 2013). Both systems were commonly used to detect small differences in sweat rate. Additionally, Smith and Havenith (2011) used the absorbent patch technique to detect regional differences in sweat rate over the entire body surface during moderate-intensity exercise, which is comparable to the current study. Therefore, it was expected that our methods were sensitive enough to detect the differences in sweat characteristics.

Apart from the physiological responses, ventilated vests could also be beneficial when reducing heat sensation. These subjective responses may apply to whole-body or local sensations. Previous research showed that local discomfort overrides whole-body comfort (Zhang et al. 2004). Thus, if the ventilated vest, a local manipulation, felt uncomfortable during the experiment, this most likely would have determined the subjective scores. However, no significant differences in subjective scores were found between conditions (Fig. 6), again implying that thermal stress was similar with and without ventilated vest.

Having such a ventilated vest one can put extra layers on top of it with a limited increase of the total thermal insulation (provided that vest inlets and outlets are not obstructed), since the vest circumvents the thermal insulation of the vest itself and layers on top. Additional tests with multiple layers (ballistic and load bearing vest; LCPB) suggest that adding an extra layer ($I_{\text{LCPB}}$ = 0.218 m^2^ KW^−1^) could increase static local thermal insulation without ventilated vest to:

$$I_{\text{system,LCPB}} = I_{\text{system}} + I_{\text{LCPB}} = 0. 2 8+ 0. 2 1 8= 0. 50 1 {\text{ m}}^{ 2} \;{\text{KW}}^{ - 1} .$$

Whereas with the inclusion of the vest (ventilated at 1.5 ms^−1^), total measured local thermal insulation was $I_{{{\text{system,LCPB}},1.5}}$ = 0.242 m^2^ KW^−1^. Given that $I_{system,1.5}$ = 0.196 m^2^ KW^−1^, wearing the LCPB only adds 0.046 m^2^ KW^−1^ instead of 0 $I_{\text{system,LCPB}}$ = 0.218 m^2^ KW^−1^. This means that the ventilated vest roughly compensates 75% of the insulation provided by additional layers. Such a ventilated vest could be beneficial in settings in which several clothing layers are worn on top of each other, for example in occupational settings (police) and the military. To be able to use such a system in contaminated environments, some practical modifications have to be made (the system allows for filters to avoid contamination by particles underneath the vest). Moreover, to verify performance, the system ideally should be evaluated according to ASTM standards (ASTM-F2300-10 2016; ASTM-F2371-16 2010; McCullough and Eckels 2009). The protocols performed in the present study diverge from these standards, especially regarding metabolic rate (~ 415–520 W (ISO8996 2004) here; 250 W prescribed by ASTM), which should be taken into account prior to usage in practice. Nevertheless, the calculations made here can potentially be used to develop a cooling regulation algorithm to predict the limit of air movement (due to walking) for which a ventilated vest is beneficial or to estimate the required ventilation to compensate for insulation of added layers. This information may be useful to extend the current verification procedures for standard settings on ventilated vest performance.

## Limitations

Findings only apply to the specific condition (34 °C, 20% RH) and exercise intensity (walking at 7 km h^−1^) that was used during the current experiment. Furthermore, as one participant was excluded from the study, the detectable effect size value increased from 0.85 to 0.90 (given *α* = 0.05, power = 0.8 and *n *= 9). This denotes we were able to detect a difference between the two conditions once the effect size exceeded 0.90, which is typically classified as a large effect. The study lacks power to detect a smaller difference.

To carry the additional mass of the ventilated vest (1.3 kg), metabolic rate could have been increased. Previously it was observed that by adding 2 kg around the waist, metabolic rate increased by 3% (Dorman and Havenith 2007). Adding 1.3 kg most likely caused a minor increase of ~ 10 W (~ 2%) in metabolic rate (Epstein et al. 1987; Pandolf et al. 1977).

It is further assumed that the underwear supported wicking of sweat, yet despite the dry conditions, at the end of the experiments the underwear was slightly wet, but the channels were dry. Therefore, on locations outside the vest that were covered by the underwear and were wet at the end of the experiment, local *E*_max_ was presumably lower than local *E*_req_. As the channels were dry, on the torso *E*_max_ > *E*_req_.

Another potential limitation could be that, as an exception due to logistical constraints, thermal insulation of the underwear ($I_{\text{uw}}$) was determined by applying wind (speed 1.5 ms^−1^) from the front. In the experiment, ventilation was applied from the front and back by the ventilated vest. As a consequence, the actual $I_{\text{uw}}$ could be slightly different. However, because the exact same underwear was worn in both conditions this is not affecting our results. Second, a ventilation speed of 1.5 ms^−1^ was achieved underneath the ventilated vest on the manikin, but due to breathing and body morphology, the actual ventilation speed during the experiment was most likely slightly lower. Therefore, additional measures with a ventilation speed of 1 ms^−1^ were performed. This resulted in $I_{{{\text{system}},r,1.0}}$ = 0.222 m^2^ KW^−1^. Presumably the dynamic $I_{{{\text{system}},r}}$ during the experiment was in between 0.222 m^2^ KW^−1^ (1 ms^−1^) and 0.185 m^2^ KW^−1^ (1.5 ms^−1^). The $I_{{{\text{system}},r}}$ value of the no-vest condition (0.188 m^2^ KW^−1^) falls within this range. Both issues may have caused an offset in our calculations.

Finally, LSR underneath the vest was not assessed in the current study for practical reasons, yet no difference underneath the vest is assumed because there was no difference in measured whole-body evaporated mass and LSR outside the vest (Fig. 3, 5). Future studies should take into account the additional measures of LSR under the vest and include recordings of wet discomfort and clinginess as they may reveal differences between both conditions.

## Conclusions

This paper shows that in two different isothermal settings, no physiological and subjective differences are observed during a fast-paced walking activity. Two clothing systems with comparable dry thermal resistance (local thermal resistance: 0.185 m^2^ KW^−1^ for the vest and 0.188 m^2^ KW^−1^ for the no-vest condition) provided equal thermal stress and local ventilation did not alter local sweating responses on body parts outside the vest. The latter can be explained by equal required evaporation, a dominant central drive for sweating and/or exercise-induced non-thermal factors. The calculations made in the current study can be used to develop a cooling regulation algorithm, predicting the limit of air movement for which a ventilated vest is beneficial and/or estimating required ventilation to compensate for the insulation of added layers, or even exceed it if applying higher ventilation rates. This could potentially be used to extend the standard verification procedures for ventilated vest performance. Such a ventilated vest could be beneficial in the military and for the police since they wear multiple clothing layers on top of each other and the ventilated vest circumvents the insulation that comes with the extra clothing.

## Abbreviations

${\text{A}}_{x}$ (m^2^): Surface area of *x*
ANOVA: Analysis of variance
Arm_L_: Left arm
Arm_R_: Right arm
BDU: Battle dress uniform
*E*_max_ (W): Maximum evaporative heat loss
*E*_req_ (W): Required evaporation for heat balance
*f*_cl,x_: Local clothing area factor of x
HR (bpm): Heart rate
$I_{x}$ (m^2^KW^−1^): Thermal insulation of *x*
$I_{x,r}$ (m^2^ KW^−1^): Resultant thermal insulation of *x*
LCPB: Ballistic and load-bearing vest
Leg_L_: Left leg
Leg_R_: Right leg
LSR (gm^−2^ h^−1^): Local sweat rate
$m_{x}$ (kg): Mass of *x*
RH (%): Relative humidity
RPE: Rating of perceived exertion
SA (m^2^): Surface area
SAM: Sweating Agile thermal Manikin
*t* (h): Time
*T*_*x*_ (°C): Skin temperature of *x*
*T*_air_ (°C): Air temperature
TC: Thermal comfort
*T*_gi_ (°C): Gastrointestinal temperature
TS: Thermal sensation
$v_{x}$ (ms^−1^): Speed of x
WBSL (kg): Whole-body sweat loss
